# Intrapulmonary mature Teratoma

**DOI:** 10.1186/1746-1596-1-38

**Published:** 2006-10-21

**Authors:** Monika Lamba Saini, S Krishnamurthy, Rekha V Kumar

**Affiliations:** 1 Department of Pathology, Kidwai Memorial Institute of Oncology, Bangalore, India; 2 Department of Surgery, Kidwai Memorial Institute of Oncology, Bangalore, India

## Abstract

Teratomas are tumors consisting of tissues derived from more than one germ cell line. Criteria for pulmonary origin are exclusion of a gonadal or other extra-gonadal primary site and origin entirely within the lung. Lung teratomas are rare, and for unknown reasons commonly involve the upper lobe of the left lung. We report a case of intrapulmonary teratoma in a 38-year-old male and review the relevant literature.

## Background

Mature teratomas are the most common histological type of germ cell tumors. These lesions originate from the third pharyngeal pouch, and may manifest with a variety of clinical and radiological features. Primary lung teratomas have rarely been reported since Mohr's description of this entity in 1839.

## Case report

A 38-year-old male presented with a two year history of intermittent episodes of cough and hemoptysis. He was a non smoker, and had no history of weight loss, fever or expectoration.

Clinical examination revealed a well-preserved young male with stable vitals. He was afebrile. Auscultation of the chest revealed coarse crackles over the left upper and middle lobes. The rest of the clinical examination was unremarkable.

**Figure 1:**
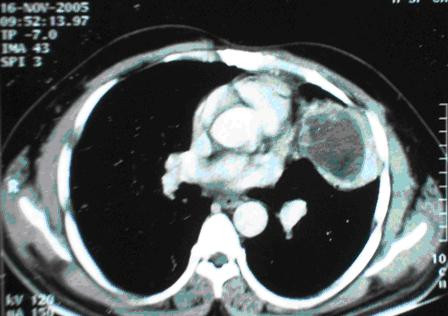
Computed tomography (CT) of the thorax showed a well defined lesion in the anterior segment of the left upper lobe.

The chest x-ray showed a well-defined large opacity in the upper lobe of the left lung. Computed tomography (CT) of the thorax showed a well defined solid lesion measuring 7 × 5.2 × 5 cm and occupying the anterior segment of the left upper lobe and the superior segment of the lingular lobe (Fig. 1). It appeared to be adherent to the left anterolateral subcostal pleura laterally and the mediastinal pleura medially, starting at the level of the main pulmonary trunk and extending along the left ventricular surface. The lesion showed heterogenous density containing soft tissue elements, fat, cystic areas, and foci of calcification, which is the classic imaging appearance of a benign teratoma. Perilesional inflammatory changes were present. No mediastinal lymphadenopathy, pleural effusion, or thickening was noted.

CT guided fine needle aspiration cytology (FNAC) showed sheets of degenerating acute inflammatory cells and anucleate squamous cells. A histological evaluation was suggested.

Pulmonary function tests showed mild restriction in the forced vital capacity. Routine hematological tests and abdominal sonography was within normal limits. Mantoux test was negative.

**Figure 2:**
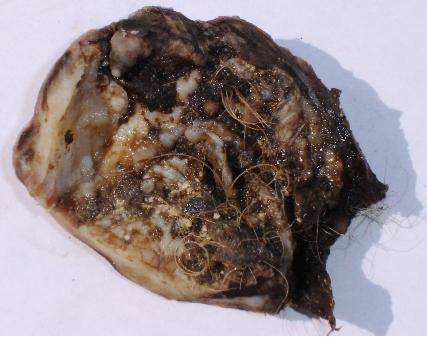
Gross surgical specimen showing encapsulated, partially cystic lesion filled with hair and sebaceous material.

**Figure 3:**
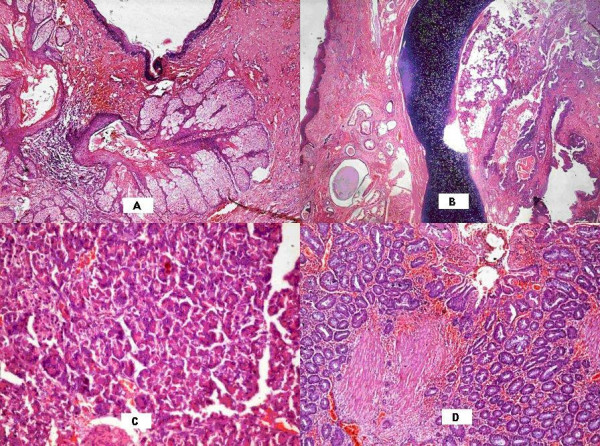
Microscopic examination showed a variety of cell lines – squamous epithelium and sebaceous glands (Fig. 3A), cartilage (Fig. 3B), pancreatic tissue (Fig. 3C), gastric glands (Fig. 3D).

Thoracotomy and enucleation of the lesion was performed. The lesion, measuring 7.5 × 6 × 5.5 cm, was well circumscribed, encapsulated, and partially cystic and filled with hair and sebaceous material (Fig 2). Microscopic examination showed a variety of cell lines (Fig. 3) consisting of squamous epithelium and sebaceous glands (Fig. 3A), cartilage (Fig. 3B), pancreatic tissue (Fig. 3C), gastric glands (Fig. 3D). Areas of cystic change, calcification, and bronchial elements were also noted.

## Discussion

Mature teratomas are the most common histological type of germ cell tumors, followed by seminomas. Germ cell tumors are predominantly found in the gonads, while the anterior mediastinum is the most common extragonadal site [1]. The first case of pulmonary teratoma was reported by Mohr in 1839 [2].

Germ cell tumors in the lung occur typically in the second to fourth decades of life with a slight female preponderance. Patients present with chest pain, hemoptysis, cough and expectoration of hair (trichoptysis); the latter is the most specific symptom [1].

Intrapulmonary teratomas typically range from 2.8 to 3 cm in diameter, and are cystic and multiloculated but may rarely be predominantly solid. In 42% of the cases, the cysts are in continuity with bronchi, and have an endobronchial component resulting in hemoptysis or expectoration of hair or sebum [3]. Microscopically, mesodermal, ectodermal and endodermal elements are seen in varying proportions. Pulmonary teratomas are mostly composed of mature, cystic somatic tissue – although malignant elements may occur. Mature elements often take the form of squamous lined cysts. Thymic or pancreatic elements may be seen in mature teratomas. Malignant pulmonary teratomas present as sarcoma or carcinoma with the presence of immature elements like neural tissue [1].

Clinically, patients with intrapulmonary teratomas present with chest pain (52%), hemoptysis (42%) and cough (39%). The most specific symptom is trichoptysis or expectoration of hair (13%). Bronchiectasis occurs in 16% of cases and may delay the recognition of the pulmonary tumor [4].

Radiographically, lesions are typically cystic masses often with focal calcification. CT accurately estimates the density of all elements such as soft tissue (in virtually all cases), fluid (88%), fat (76%), calcification (53%) and teeth [5]. MRI is valuable in detecting the anatomic relation to mediastinal and hilar structures.

Surgical resection is the treatment of choice; and radical extirpation leads to a long recurrence-free survival [6].

## Conclusion

Intrapulmonary teratomas are rare tumors. They originate from the third pharyngeal pouch and present as cystic lesions in the majority of cases. Histologically, benign teratomas comprise 2 or 3 primordial layers. Patients present with chest pain, cough, hemoptysis and trichoptysis. Complete resection is adequate treatment for patients with a good long term prognosis.
